# Ante-Mortem Clinical Characterization with Post-Mortem Family Interview and Medical Record Abstraction in a Traumatic Brain Injury Brain Donor Program

**DOI:** 10.1177/08977151251362180

**Published:** 2025-08-05

**Authors:** Amelia J. Hicks, Enna Selmanovic, Ariel Pruyser, Carley R. Trentman, Joshua C. Klein, Kaitlyn Wilkey, Miguel X. Escalon, Natalia Bernal-Fernández, Belinda Yew, Kristen Dams-O’Connor

**Affiliations:** ^1^ Department of Rehabilitation and Human Performance, Icahn School of Medicine at Mount Sinai, New York, New York, USA.; ^2^ Nash Family Department of Neuroscience, Icahn School of Medicine at Mount Sinai, New York, New York, USA.; ^3^ Friedman Brain Institute, Icahn School of Medicine at Mount Sinai, New York, New York, USA.; ^4^ Department of Neurology, Icahn School of Medicine at Mount Sinai, New York, New York, USA.

**Keywords:** post-mortem family interview, TBI, traumatic brain injury, verbal autopsy

## Abstract

Recent investments in large-scale mortem tissue collection have accelerated opportunities to understand the neuropathology of traumatic brain injury (TBI) and post-traumatic neurodegeneration (PTND). Clinicopathological correlation requires ante-mortem clinical information. Post-mortem family interviews (PFIs) are an established method to capture comprehensive ante-mortem clinical information. The aim of this report was to summarize our experience of using the PFI in the Late Effects of TBI (LETBI) brain donor program to facilitate replication, expansion, and refinement of PFI methods in TBI brain donor programs. We describe the content development and structure of the LETBI PFI; interviewer training and qualifications; and considerations regarding interview duration, informant selection, and interview timing, as well as PFI inter-rater reliability. We also compare the information captured in the PFI with data abstracted from the medical records for 34 decedents in the LETBI brain donor program to illustrate the complementarity of these approaches and highlight the unique contributions of the PFI. The PFI can provide granular details about a decedent’s clinical history and symptom trajectories over time, potential contributing factors to PTND including social determinants of health (e.g., race and years of education), family history of medical and psychiatric conditions, and contextual information regarding cause(s) of death. The PFI is an important component of a multi-modal autopsy that provides unique insights essential for clinical–pathological correlation investigations of chronic TBI and PTND neuropathology.

Converging evidence suggests that approximately one-third of traumatic brain injury (TBI) survivors will experience progressive decline in the chronic period that is not accounted for by age.^1–3^ Trajectories of clinically meaningful decline have been identified in cognitive,^3,4^ neurobehavioral,^5^ motor,^6^ and functional^2,7^ outcomes. The term post-traumatic neurodegeneration (PTND) has recently been employed to define this decline^8–13^ and distinguish it from the chronic but stable impairments expected after TBI. PTND is considered a multiple/mixed etiology dementia,^14^ with multiple pathological processes implicated.^15–18^ Preliminary evidence suggests PTND clinical phenotypes may be distinguishable from Alzheimer’s disease^19^ and all-cause dementia.^20^ Associations between clinical symptoms and underlying pathologies remain poorly understood, making the investigation of well-characterized clinical cohorts with autopsy end-points a research priority.^11,12^

Historically, post-mortem tissue was only available for individuals who had died acutely after TBI.^21–33^ Now, brain tissue is increasingly available in large chronic TBI cohorts,^34,35^ creating opportunities to identify mechanistic pathological processes involved in chronic TBI and PTND. Clinicopathological correlations, the process of identifying associations between clinical symptoms observed during life and pathological changes observed in post-mortem brain tissue, rely on the availability of ante-mortem clinical data. However, comprehensive ante-mortem data are only available for the extreme minority of cases enrolled in longitudinal studies with autopsy end-points. Efforts to identify common data elements (CDEs) for post-mortem characterization of ante-mortem clinical functioning in decedents with TBI suggest that even minimal information can be instructive.^36^ Post-mortem family interviews (PFIs) provide a well-established^37–45^ method for obtaining comprehensive ante-mortem data to characterize clinical symptoms.

PFIs, also known as verbal autopsies, have been used by clinical researchers for decades.^46^ PFIs are typically conducted with people close to the decedent and are designed to characterize cause of death, trajectories of health and function, and care prior to death.^46–51^ The content, structure, and duration of the PFIs are flexible to the clinical population and aims of use. Historically, PFIs have played a central role in determining the cause of death in settings where the cause of death and forensic autopsy are unavailable or infeasible.^46,49,50^ As the value of PFIs in clinical research has been increasingly acknowledged, their use has expanded beyond the cause of death determinations.^49^ PFIs have become a primary source of information to inform ante-mortem diagnosis of psychiatric and neurological conditions, including dementia,^37–40^ to identify precipitants of suicide,^41,43^ and to characterize ante-mortem cognitive, neurobehavioral, and psychological functioning.^37,44,45^ Notably, PFIs are used as a primary source of information about cause of death and clinical disease progression in several large U.S. federally funded studies.^34,35,52–55^

The PFI provides a unique opportunity to obtain information traditionally overlooked by the medical record (MR) or death certificate.^49,56^ This includes clinical symptoms and care in the months, weeks, and days prior to death,^44^ systemic and environmental contributors to death,^49,56^ and information about cultural understandings of disease and explanations of death.^56^ PFIs may be particularly informative for decedents with limited health care utilization.^48,56^ This includes marginalized groups known to experience barriers to health care, such as those within the LGBTQIA+ community^57^ and people with disabilities.^58,59^

Evaluations of PFIs have focused on consistency and comparability with other measures (e.g., MRs) and the usefulness of the PFI in filling important knowledge gaps.^49,60^ Assessment of the absolute validity of PFIs has been discouraged, as no gold standard is available for comparison.^49,60^ It has long been recognized that information provided in death certificates is often missing, incomplete, or innacurate,^61,62^ and the quantity and quality of information contained in MRs have been consistently scrutinized.^49^ In this context, it is not surprising that one study found PFIs had higher specificity and sensitivity than death certificates when used to determine cause of death.^63^ This may also reflect the flexibility of the PFI to capture multiple contributing causes of death.^49^

Reliance on MRs may be particularly problematic for the TBI population. Many patients who sustain TBI may not present for medical care (e.g., sports concussion and victims of intimate partner violence) and will therefore not have TBI in their MR.^64,65^ Contrary to expectations, individuals with undiagnosed/untreated TBIs have cognitive and functional impairments that are similar to or worse than those with diagnosed/treated TBIs.^66,67^ Furthermore, many patients will present only to outpatient settings and will be missed if only hospital records are relied upon.

PFIs have recently been incorporated into large prospective studies of repetitive head impacts^34,35^ and TBI^34^ to inform clinicopathological correlations. Our team has led one of these efforts developing and implementing a PFI as part of the Late Effects of TBI (LETBI)^34^ study. LETBI is a prospective brain donor program that aims to elucidate the clinical signatures and biological mechanisms of PTND. To diversify the LETBI brain archive, participants with no ante-mortem data can be enrolled at the time of their death with consent from their next of kin. To our knowledge, LETBI is the first study to include a multi-modal autopsy for survivors of TBI that includes PFI with informants and MR review, in addition to *ex vivo* neuroimaging and comprehensive image-guided neuropathological examination. This report summarizes our experience of using a PFI in the LETBI brain donor program to facilitate replication, expansion, and refinement of PFI in TBI brain donor programs.

## LETBI PFI Content Development and Structure

The purpose of the LETBI PFI is to gather objective and subjective information regarding the decedent’s developmental history, head trauma exposure, medical health and medications, family history of psychiatric and neurological conditions, functional independence, sleep, substance use history, lifetime cognitive, motor, emotional, and neurobehavioral function, and the circumstances surrounding their death. The LETBI PFI is a semi-structured questionnaire (Table 1) that includes validated measures and items informed by the Reasons for Geographic and Racial Differences in Stroke study,^53,69^ the Midlife in the United States study,^54,70^ the National Alzheimer’s Coordinating Center,^55,71,87^ the Understanding Neurologic Injury and Traumatic Encephalopathy study,^35,72^ Adult Changes in Thought,^79^ and the TBI Model Systems^77,88^ to facilitate comparisons. The measures were adapted as needed to be appropriate for the informant report and the setting of chronic TBI. The items include but dramatically expand upon the National Institute of Neurological Disorders and Stroke *in vivo*^89^ and post-mortem^36^ TBI CDEs.

**Table 1.** Measures Included in the LETBI PFI

Measure	Brief description	Modification for informant report	Reference	Post-mortem TBI common data elements
Introductory questions	A series of open-ended questions covering birth and developmental milestones, early childhood—family life, scholastic achievements, hobbies, employment history, relationship history and family composition, friendships, and support network during adulthood.	N/A	LETBI team generated items designed to approximate methods of a clinical intake interview and develop rapport with informant(s).	N/A
Brain Injury Screening Questionnaire (BISQ)	A structured questionnaire that characterizes prevalence and severity of lifetime exposure to head trauma and TBI. The BISQ includes 20 contextual recall cues (e.g., in a vehicular accident, falling from a height, drug or alcohol blackout, diving into water). For each reported exposure, subsequent items record the presence and duration of loss of consciousness (LOC) and/or altered mental status (AMS) and date of injury.	Item wording was changed to facilitate informant report: “Have you ever experienced a blow to the head from….” > “Has (decedent name) ever experienced a blow to the head from….” As per the standard self-report BISQ, informants are encouraged to provide their best estimate for date of injury, with the code “01/01/YEAR” used for injuries in which only the year is known. Likewise, to maximize data captured, informants may endorse the presence of unconsciousness but respond “Don’t Know” for duration.	Dams-O’Connor et al. (2014)^67^	Core
BISQ Sports and Military modules	A structured questionnaire that characterizes lifetime exposure to repetitive head impacts sustained in the context of organized sports and military service. Six specific sports are queried (hockey, tackle football/American football, lacrosse, rugby, soccer/association football, boxing/combat sports/wrestling, and MMA) with an option to provide an unlisted sport. Subsequent items record ages of sports play, highest league/division, and blows to the head (date, presence, and duration of LOC and AMS). Military items include age of entry and exit to military, branch, highest rank, years of combat training, deployment, and combat, boxing training, exposure to blast, blows to the head (date, presence and duration of LOC and AMS).	Item wording to facilitate informant reports, and options to respond “Don’t Know” are as per BISQ (above).	LETBI team generated items in partnership with colleagues as part of NIH 1U01NS086659-01 and 1U01NS086625 with guidance from existing tools.^68^	Core
BISQ Intimate Partner Violence (IPV) module	A structured questionnaire developed in partnership with IPV survivors to characterize lifetime exposure to head trauma and nonfatal strangulation/suffocation events sustained in the context of IPV. Seven contextual recall cues are provided (e.g., pushed/shoved, broken teeth or jaw, eye or ear injuries) with subsequent items querying date, presence, and duration of LOC and AMS.	Item wording to facilitate informant reports, and options to respond “Don’t Know” are as per BISQ (above).	Dams-O’Connor et al. (2023)^11^	Supplemental—highly recommend
Demographics	A structured questionnaire that captures demographic details of the informant and decedent, including age, sex, race, education, and employment.	N/A	Informed by REGARDS,^69^ MIDUS^70^	Core^a^
Participant death information	The measure includes both single-item questions (e.g., cause of death, place of death, health conditions present in the months PTD, care sought) and semi-structured interview-style questions that explore illness trajectory over the final years, months, and days preceding death (e.g., “When you look back, do you think there were other/ early signs that [the decedents’ health was declining in the year before they died? What were some things you noticed?)”. Where a death certificate has been issued, we record the immediate, underlying, and contributing causes of death from the certificate.	N/A	Informed by REGARDS^69^	Core^a^
NACC Form B9: Clinical Judgment of Symptoms	Characterizes cognitive, behavioral, and motor decline in the year PTD relative to previously attained abilities. Each domain includes several subdomains/symptoms of function (e.g., the behavior domain includes apathy, delusions, and agitation), age and mode of onset, and predominant symptom. The interviewer uses clinical judgment to determine item responses based on the informant’s answers. For example, if the informant reports memory impairment that upon probing is more consistent with attentional dysfunction, the interviewer will record the presence of “attention and/or concentration” problems. Two items capture the informants’ perspectives directly: (1) whether the informant identified memory decline PTD and (2) whether the decedent had self-identified memory decline PTD.	N/A	Informed by NACC^71^	Core
Cognitive and motor function	Measure of difficulties with immediate and delayed memory, attention, language, problem solving, spatial skills, and motor function (e.g., shuffling gait, rigidity, slowness). Records age of onset for each endorsed impairment.	N/A	Informed by UNITE^72^	Core
Cognitive Difficulties Scale	38 item measures assessing difficulties in functional cognition, such as difficulty naming objects and recognizing familiar faces. Degree of difficulty with each task rated from 1 “Very often” to 5 “Not at all”.	N/A	McNair et al. (1983)^73^	Core
Mental State Evaluation (IQCODE)	16 items requiring the informant to judge whether there was a change in their loved one’s cognitive function in the last few months PTD as compared with 1-year PTD.	N/A	Jorm et al. (1988)^74^ (modified)	Core
Neuropsychiatric Inventory Questionnaire	Covers 12 domains of neuropsychiatric function including delusions, anxiety, depression or dysphoria, and disinhibition. For each domain, informants report the age symptoms began, the severity of the symptoms on a 3-point scale (mild, moderate, and severe), and the course of symptoms (stable, worse, improve, and fluctuate).	N/A	Cummings et al. (1994, 2020)^75,76^ (modified) Used in UNITE^72^	Core
Psychosocial problems	History of law violations, psychiatric hospitalizations, and suicide attempts. All items are queried for the time period before and after head injury exposure and answered on an item-specific Likert scale.	TBIMS items are routinely used with informants, with whom item wording specifies, “Has (name) ever been accused of breaking the law?”.	Informed by TBINDSC^77^	Supplemental—highly recommend^a^
Mayo Sleep Questionnaire	Screening measure for sleep problems. Only items for RBD and OSA are included. Items query formal diagnoses and potential signs observed by the informant (e.g., “Did [decedent] ever seem to stop breathing during sleep?”).	N/A	Boeve et al. (2011)^78^ (modified)	Supplemental—highly recommend^a^
Family history	History of neurological and psychiatric disorders in first-degree family members.	N/A	Informed by REGARDS,^69^ MIDUS,^70^ NACC,^71^ UNITE,^72^ ACT^79^	
Revised Amyotrophic Lateral Sclerosis Functional Rating Scale (ALSFRS-R)	Characterizes symptoms of ALS by comparing functioning in the last month of life and prior to ALS symptom onset.	The introductory statements of this measure were altered to replace reference to “your function” with “(decedent’s) function.”	Cedarbaum et al. (1999)^80^ (modified per UNITE^72^)	Core^a^
Disease Burden Morbidity Assessment	Queries the ADL burden consequent to medical conditions. Used the modified 21-item version and includes an additional item for depression and anxiety (i.e., 22 items total).	Item wording was changed to facilitate informant reports, with all items using the structure “Did (the decedent) suffer from (condition)?”.	Bayliss et al. (2009)^81^; Poitras et al. (2012)^82^	Core
Health history	Lists 29 health conditions and asks the respondent to record whether they were present in the year and month PTD.	The introductory statements of this measure were altered to replace “…did you experience…” to “did (the decedent) experience…”	Informed by MIDUS^70^	Core^a^
Medications	Medications prescribed in the year and month of PTD. Medication name and reason for prescription recorded for every medication.	N/A	Informed ACT^79^	Supplemental—highly recommend^a^
Hospitalizations/surgeries in the year prior to death	All hospitalizations (from physical health problems) and surgeries requiring general anesthesia in the year PTD.	N/A	Informed by REGARDS,^69^ MIDUS^70^	Core^a^
NACC Functional Activities Questionnaire	Measures instrumental activities of daily living (IADLs), such as preparing balanced meals and managing personal finances. The informant is asked whether the decedent had difficulties in 10 IADL tasks in the 4 weeks PTD. Response options are “Not Applicable (i.e., never did),” “Normal,” “Has difficulty, but does by self,” “Requires assistance,” and “Dependent.”	N/A	Informed by NACC^71^	Core
SF-36 Health Survey: Physical Functioning Sub-scale	Examines functional impact of decedent’s health problems on everyday physical function in the year PTD. The informants asked whether there was a change in 10 physical activities, including “Lifting or carrying groceries” and “Walking several blocks.” Responses were provided on a 4-point Likert scale from “Major Decrease” to “Some Improvement.”	The introductory statements of this measure were altered to replace references to “your health problems”/ “your ability to do…” with “(decedent’s) health problems,” etc.	Ware et al. (1992)^83^	Supplemental
Functional Assessment Staging of Dementia of the Alzheimer’s type (FAST)	The FAST scale outlines seven distinct stages of the functional decline in Alzheimer’s disease (AD). Stages 1 and 2 represent the functional ability of an adult without AD, and stage 7 represents functional ability of an adult in the final and most severe stage of the disease. Each stage includes one or more symptoms (e.g., Stage 6—urinary incontinence; Stage 7—unable to smile), for which the duration of symptoms is also recorded.	N/A	Reisberg (1988)^84^	
Assessment of Clinical Frailty	Reviews the presence of 15 frailty indicators in the years PTD and records the onset date. Frailty indicators include sarcopenia, muscle weakness, risk of falls, unintentional weight loss, and frequent viruses.	N/A	Informed by NACC^71^ and ACT^79^	
WHO ASSIST V3.0	Records whether the decedent has ever tried alcohol, tobacco, and nine drugs, including opioids, stimulants, and hallucinogens.	Item wording was changed to facilitate informant report: “In your life, which of the following substances have you ever used?” > “In (decedent’s) life, which of the following substances have they ever used?”.	WHO ASSIST Working Group (2002)^85^ (modified)	Core^a^
Tobacco use	Records frequency and amount of tobacco intake, as well as age when tobacco intake ceased (if relevant).	Item wording was changed to facilitate informant report, with “you” changed to “(decedent name)” for all items—e.g., “If (decedent name) quit smoking, at what age did they quit?”.	Informed by MIDUS^70^	Core^a^
Brown-Goodwin Assessment for Lifetime History of Aggression	12 items measuring aggressive and antisocial behaviors throughout an individual’s life. Data were taken at three time points: childhood, adolescence, and adulthood. For all endorsed items, the informant is asked to specify when the incident occurred and provide a brief description.	N/A	Brown et al. (1979)^86^	
Lifestyle questions	Open-ended items exploring the following lifestyle domains in the months and years prior to death: social/community involvement, exercise, cognitive stimulation, diet.	N/A	Original to LETBI PFI. researcher generated items	

^a^ Adapted TBI post-mortem CDE—key constructs overlap. Measures and items were modified to (1) allow for informant report on traditionally self-report measures, (2) to refer to a specific timeframe prior to death, and/or (3) to specify “relative to highest level of post-injury function” to distinguish PTND from post-TBI chronic stable impairment.

Beekly et al., 2004^55^, 2007^87^.

ACT, adult changes in thought; AD, Alzheimer’s disease; ADL, activities of daily living; ALS, amyotrophic lateral sclerosis; IADLS, instrumental activities of daily living; LETBI, late effects of traumatic brain injury; MIDUS, midlife in the United States; NACC, National Alzheimer’s Coordinating Center; OSA, obstructive sleep apnea; PFI, post-mortem family interview; PTD, prior to death; RBD, rapid eye movement sleep behavior disorder; REGARDS, reasons for geographic and racial differences in stroke; TBIMS, Traumatic Brain Injury Model Systems; UNITE, Understanding Neurologic Injury and Traumatic Encephalopathy.

### Interviewer training and qualifications

Our interviewer team included post-doctoral fellows and senior faculty with training in clinical neuropsychology and clinical research coordinators. All interviewers were required to be familiarized with the interview content, listen to ≥2 recorded interviews, and shadow ≥1 interview. Peer supervision was provided monthly by a senior psychologist. Non-clinical research staff required some additional training in sensitive topics, such as bereavement and trauma, and close supervision from a clinician.

### Duration of interview

The LETBI PFI requires approximately 2–3 h to complete. Decedents with a longer illness course, more TBI exposures, multiple informants, and/or a complex psychosocial history can require a longer PFI.

### Types of informants

Informants have included parents, partners, siblings, adult children, friends, and other relatives. The type of informant can impact the information provided in a PFI. Partners and some adult children can provide detailed information about decedents’ day-to-day lives, including details about the onset and course of clinical changes. However, they may struggle to provide details about early life, which can impact TBI exposure ascertainment, as well as differentiation of premorbid neurobehavioral functioning from TBI- or disease-related change. Parent-informants tend to be well-informed about head trauma exposure and the chronology of clinical symptoms with onset in earlier life. In general, older informants may have difficulty recalling specific details. Interviewing friends, teammates, and/or “battle buddies” is highly informative for capturing head trauma and symptom course in the context of intimate partner violence, contact sports, military service, or life stages with which family members may be less familiar.

### Timing of interviews

Scheduling interviews at an appropriate time after death requires careful consideration on a case-by-case basis with the informants’ comfort always prioritized. Conducting interviews within weeks to months of death can optimize informants’ recall of specific details leading up to death and support the grieving process. However, this period can also be characterized by raw emotions and/or the overwhelming logistical sequelae that may follow a loved ones’ death. Although later interviews could compromise accuracy, informants have had more time to gather information from others (e.g., friends and police reports), process the bereavement, and reflect on clinically relevant changes.

## Comparison of Missingness in PFI and MR Abstraction

All study procedures were approved by the Icahn School of Medicine at Mount Sinai program for the protection of human subjects.

We compared the information captured in the LETBI PFI with MR for a consecutive series of 34 decedents. The data extraction form was limited to PFI domains that could be captured in the MR (e.g., demographic information, medical history, and clinical symptoms); domains such as leisure activities and early life psychosocial history were omitted. Four physician team members (M.X.E., K.W., J.C.K., and C.R.T.) were trained by the research team (K.D.O.C. and E.S.) in data extraction procedures. Each PFI or MR was extracted independently by two of the four raters. No rater reviewed both the PFI and MR for the same case (i.e., data sources were counterbalanced to minimize bias). One team member (A.J.H.) reviewed all four extractions for each decedent. Data were considered missing when an item was not captured in either extraction. The frequency of missingness was compared for each data point across sources. Open-ended responses (e.g., descriptions of surgery) in the extractions were compared qualitatively by two team members (A.J.H. and E.S.).

### Inter-rater reliability of PFI abstraction

We compared the concordance between data abstracted by two independent raters for each PFI (*n* = 34 decedents). An item was considered concordant when, based on open text fields and structured questionnaire responses, both data abstractors extracted the same data for a given data element (e.g., both extractors agreed that memory impairment was present) or when both team members agreed the data point was missing.

## Results

### Missingness comparison

#### Informant relationships and interview timing

Informants were mostly partners (*n* = 14, 41%) or adult children (*n* = 11, 32%; other family member or care giver *n* = 7, 21%; friend *n* = 2, 6%). Several informants cohabited with the decedent (*n* = 10, 29%); for those who lived separately, the frequency of contact with the decedent was usually daily (*n* = 14, 41%) or weekly (*n* = 8, 24%), and rarely monthly (*n* = 1, 3%) or no contact in the past year (*n* = 1, 3%). The average time between death and the PFI was approximately 9 months (range 2 weeks–3 years).

#### Decedent demographics

Both sources included date of birth, date of death, and gender for all decedents (Table 2). Across other demographic items, the frequency of missingness was higher for the MR, including race (59%), ethnicity (74%), country of birth (82%), and years of education (71%).

**Table 2.** Data Items Captured in the Post-Mortem Family Interview and Medical Record for *n* = 34 Decedents

	Frequency of missingness *n* (%)		Frequency of missingness *n* (%)	
	Post-mortem family interview	Medical record	Missing in PFI; endorsed as present in MR	Missing in MR; endorsed as present in PFI
Demographics				
Date of birth	0	0	N/A	N/A
Multiple birth	4 (12%)	25 (74%)	0	3 (9%)
Date of death	0	0	N/A	N/A
Gender	0	0	N/A	N/A
Race	0	20 (59%)	N/A	N/A
Ethnicity	1 (3%)	25 (74%)	N/A	N/A
Marital status	0	3 (9%)	N/A	N/A
Residence	1 (3%)	13 (38%)	N/A	N/A
Type of residence	1 (3%)	7 (21%)	N/A	N/A
Primary caregiver	0	8 (24%)	N/A	N/A
Subject lived with	1 (3%)	5 (15%)	N/A	N/A
English as primary language	0	8 (24%)	N/A	N/A
Country of birth	0	28 (82%)	N/A	N/A
Years of education	0	24 (71%)	N/A	N/A
Primary occupation	1 (3%)	13 (38%)	N/A	N/A
Details of death				
Cause of death	4 (12%)	26 (76%)	N/A	N/A
Place of death	0	24 (71%)	N/A	N/A
Medical health and medications				
Surgeries YPTD	6 (18%)	3 (9%)	6 (18%)	3 (9%)
Hospitalizations YPTD	1 (3%)	10 (29%)	1 (3%)	10 (29%)
Medications YPTD	3 (9%)	1 (3%)	3 (9%)	1 (3%)
Family medical and psychiatric history				
Memory problems	5 (15%)	19 (56%)	0	3 (9%)
Developmental disability	3 (9%)	21 (62%)	0	2 (6%)
Neurological disorders	4 (12%)	18 (53%)	0	2 (6%)
Psychiatric problems	4 (12%)	20 (59%)	0	4 (12%)
Clinical function				
Decline in clinical function noted by a clinician	11 (32%)	18 (53%)	5 (15%)	8 (24%)
Overall course of clinical decline	8 (24%)	22 (65%)	N/A	N/A
Predominant domain of impairment/decline	8 (24%)	21 (62%)	N/A	N/A
Clinical function—cognitive dysfunction				
Memory impairment present	1 (3%)	1 (3%)	0	0
Remembered things about family and friends (e.g., occupations, birthdays)	16 (47%)	25 (74%)	4 (12%)	6 (18%)
Remembered things that happened recently (e.g., friends visiting)	15 (44%)	25 (74%)	4 (12%)	7 (21%)
Recalled conversations from a few days earlier	15 (44%)	25 (74%)	4 (12%)	6 (18%)
Remembered their address/phone number	16 (47%)	25 (74%)	4 (12%)	5 (15%)
Remembered the day and month	15 (44%)	25 (74%)	4 (12%)	6 (18%)
Remembered where things were usually kept (e.g., milk is in the refrigerator)	15 (44%)	25 (74%)	4 (12%)	6 (18%)
Remembered where to find things which had been put in a different place than usual	16 (47%)	25 (74%)	4 (12%)	5 (15%)
Knew how to work familiar machines around the house (e.g., the toaster)	16 (47%)	25 (74%)	4 (12%)	5 (15%)
Learned how to use a new gadget or machine around the house	17 (50%)	25 (74%)	4 (12%)	5 (15%)
Learned new things in general	19 (56%)	25 (74%)	5 (15%)	5 (15%)
Executive function impairment present	1 (3%)	4 (12%)	1 (3%)	2 (6%)
Made decisions on everyday matters	16 (47%)	25 (74%)	5 (15%)	7 (21%)
Handled other everyday arithmetic problems (e.g., knew how much food to buy)	18 (26%)	25 (74%)	6 (18%)	7 (21%)
Used his/her intelligence to understand what was going on and to reason things out	17 (50%)	24 (71%)	6 (18%)	7 (21%)
Language	1 (3%)	3 (9%)	1 (3%)	1 (3%)
Ability to speak limited (1–5 words a day)	19 (56%)	24 (71%)	2 (6%)	5 (15%)
All intelligible vocabulary lost	22 (65%)	22 (65%)	3 (9%)	2 (6%)
Visuospatial function impairment present	2 (6%)	6 (18%)	0	0
Attention impairment present	1 (3%)	6 (18%)	0	4 (12%)
Followed a story in a book or on TV	16 (47%)	25 (74%)	5 (15%)	6 (18%)
Fluctuating cognition present	1 (3%)	11 (32%)	0	5 (15%)
Predominant cognitive symptom	2 (6%)	7 (21%)	N/A	N/A
Mode of onset of cognitive symptoms	1 (3%)	5 (15%)	N/A	N/A
Clinical function—neurobehavioral and emotional dysfunction				
Apathy	3 (9%)	26 (76%)	0	9 (26%)
Depression	4 (12%)	23 (68%)	1 (3%)	9 (26%)
Visual hallucinations	5 (15%)	27 (79%)	0	5 (15%)
Auditory hallucinations	5 (15%)	26 (76%)	2 (6%)	1 (3%)
Disinhibition	5 (15%)	27 (79%)	1 (3%)	3 (9%)
Irritability	5 (15%)	22 (65%)	0	10 (29%)
Agitation	4 (12%)	18 (53%)	0	6 (18%)
Personality change	5 (15%)	24 (71%)	1 (3%)	5 (15%)
Rapid eye movement sleep behavior disorder	7 (12%)	27 (79%)	1 (3%)	4 (12%)
Anxiety	18 (53%)	21 (62%)	4 (12%)	0
Predominant neurobehavioral/emotional symptom	13 (38%)	26 (76%)	N/A	N/A
Mode of onset of neurobehavioral/emotional symptom	13 (38%)	25 (74%)	N/A	N/A
Clinical function—motor impairment				
Gait disorder	3 (9%)	14 (41%)	3 (9%)	6 (18%)
Falls	3 (9%)	21 (62%)	1 (3%)	8 (24%)
Tremor	4 (12%)	23 (68%)	0	6 (18%)
Slowness	4 (12%)	25 (74%)	2 (6%)	16 (47%)
Predominant motor symptom	10 (29%)	24 (71%)	N/A	N/A
Mode of onset of motor symptom	9 (26%)	23 (68%)	N/A	N/A
Lifting or carrying groceries	1 (3%)	23 (68%)	1 (3%)	8 (24%)
Bathing or dressing themselves	1 (3%)	23 (68%)	1 (3%)	13 (38%)
Climbing several flights of stairs	1 (3%)	24 (71%)	1 (3%)	12 (35%)
Climbing one flight of stairs	1 (3%)	24 (71%)	1 (3%)	11 (32%)
Bending, kneeling or stooping	1 (3%)	23 (68%)	1 (3%)	12 (35%)
Walking more than one mile	1 (3%)	24 (71%)	1 (3%)	13 (38%)
Walking several blocks	1 (3%)	24 (71%)	1 (3%)	10 (29%)
Walking one block	1 (3%)	24 (71%)	1 (3%)	11 (32%)
Vigorous activity (e.g., running)	2 (6%)	24 (71%)	1 (3%)	11 (32%)
Moderate activity (e.g., bowling)	2 (6%)	23 (68%)	1 (3%)	9 (26%)
Non-ambulatory	16 (47%)	19 (56%)	5 (15%)	7 (21%)
Unable to sit up independently	19 (56%)	21 (62%)	5 (15%)	6 (18%)
Unable to smile	25 (74%)	27 (79%)	1 (3%)	2 (6%)
Unable to hold head up	24 (71%)	26 (76%)	1 (3%)	5 (15%)
Clinical function—functional impairment				
Urinary incontinence	18 (53%)	20 (59%)	4 (12%)	6 (18%)
Fecal incontinence	20 (59%)	26 (76%)	2 (6%)	8 (24%)
Simple financial management (e.g., paying bills)	4 (12%)	19 (56%)	3 (9%)	12 (35%)
Handled money for shopping	17 (50%)	25 (74%)	5 (15%)	6 (18%)
Complex financial management (e.g., tax filing)	4 (12%)	19 (56%)	3 (9%)	11 (32%)
Handled financial matters (e.g., their pension, dealings with the bank)	17 (50%)	25 (74%)	5 (15%)	5 (15%)
Shopping	5 (15%)	19 (56%)	4 (12%)	14 (37%)
Hobbies and skilled games (e.g., chess)	7 (21%)	18 (53%)	4 (12%)	9 (26%)
Basic household tasks (e.g., make a cup of coffee)	6 (18%)	19 (56%)	5 (15%)	11 (32%)
More complex household tasks (e.g., cooking)	6 (18%)	18 (53%)	5 (15%)	10 (29%)
Tracking current events	6 (18%)	21 (62%)	4 (12%)	10 (29%)
Understanding TV/book	6 (18%)	2 (6%)	5 (15%)	11 (32%)
Remembering appointments/holidays/medications	6 (18%)	21 (62%)	3 (9%)	14 (41%)
Traveling outside neighborhood—driving/public transport	7 (21%)	18 (53%)	5 (15%)	12 (35%)
Decreased ability to perform complex tasks (e.g., planning dinner for guests)	19 (56%)	23 (68%)	6 (18%)	7 (21%)
Requires assistance in choosing proper clothing	22 (65%)	22 (65%)	8 (24%)	4 (12%)
Difficulty putting clothing on properly	18 (53%)	20 (59%)	7 (21%)	4 (12%)
Substance use				
Tobacco products	0	5 (15%)	0	2 (6%)
Alcoholic beverages	2 (6%)	6 (18%)	1 (3%)	4 (12%)
Cannabis	29 (85%)	15 (44%)	2 (6%)	2 (6%)
Cocaine	31 (91%)	15 (44%)	1 (3%)	0
Amphetamine type stimulants	31 (91%)	15 (44%)	1 (3%)	0
Inhalants	31 (91%)	15 (44%)	1 (3%)	0
Sedatives or sleeping pills	30 (88%)	15 (44%)	1 (3%)	0
Hallucinogens	31 (91%)	15 (44%)	1 (3%)	0
Opioids	31 (91%)	14 (41%)	3 (9%)	0

MR, medical record; PFI, post-mortem family interview; YPTD, year prior to death.

#### Details of death

The MR did not capture the place of death (i.e., hospital and home) for 76% of decedents, while this was recorded in all PFIs. Likewise, the MR did not list the cause of death for 76% of decedents, while PFI missingness was only 12%. The PFI commonly listed conditions that were directly related to death as well as contributions from longstanding conditions and circumstantial contributors. For example, an MR indicated “aspiration pneumonia,” while PFI included “fall, trouble breathing, aspiration pneumonia” and also noted that insurance coverage for physical therapy was discontinued, resulting in several falls, prolonged hospitalization, and pneumonia.

#### Medical history

Medical history in the year prior to death was well documented by both sources. Missingness was low for the presence of medications in both sources (PFI 9% vs. MR 3%). However, more medications were typically listed in the MR. Although both sources typically included the specific medication name, informants sometimes reported the indication instead (e.g., “chemo meds” and “seizure meds”). The MR was more likely to be missing hospitalizations (29% vs. PFI 3%), whereas the PFI was more likely to be missing surgeries (18% vs. MR 9%). The MR was more likely to provide detailed terminology for surgeries, while the PFI included general reasons for a surgery and the organ operated on.

#### Family medical and psychiatric history

The PFI had limited missingness on all family medical and psychiatric history items (9–15%) compared with 53–62% missingness on the MR. Between 6% and 12% of decedents missing family history data on the MR had a positive family history according to the PFI. A positive family history on MR was never missed in the PFI.

#### Clinical symptoms

The MR was more likely to be missing information about the presence of clinical decline (53% vs. PFI 32%), the course of decline (65% vs. PFI 24%), and the predominant domain of decline (62% vs. PFI 24%). Notably, 24% of decedents who had no indication of clinical decline in the MR had clinical decline noted on the PFI. Examining the open-ended items, the age of decline onset in the MR was usually later.

The rates of missingness for both sources on the presence of cognitive impairment (e.g., memory impairment and executive dysfunction) were very low (3% PFI vs. 3–12% MR). However, the MR was often missing more granular descriptions of memory impairment (71–74% vs. 26–56% PFI). A similar pattern of missingness was noted for the overall presence of language impairment (3% PFI vs. MR 9%); however, the sources had similar levels of missingness for detailed items (56–65% PFI vs. 65–71%). For attentional dysfunction, fluctuating cognition, and visuospatial impairment, the MR had more decedents with missing information (18–32% MR vs. 3–6% PFI). The MR was missing neurobehavioral and/or emotional dysfunction information for 62–79% of decedents, as compared with only 9–53% of decedents on the PFI. For motor impairment, the PFI had much less missingness (3–29% vs. MR 41–71%). There were, however, similar rates of missingness on four motor-related items (PFI 47–74% vs. MR 56–79%): (1) non-ambulatory, (2) unable to sit up independently, (3) smile, or (4) hold head up. The rates of missingness on both sources were more variable for functional impairment (PFI 18–65% vs. MR 6–76%).

#### Substance use

The rates of missingness for alcohol and tobacco intake were higher, albeit still low in the MR (15–18% vs. PFI 0–6%). In comparison, the MR had less missingness for all other substances (41–44% vs. PFI 85–91%). Examining the open-ended responses, the PFI recorded higher doses for both alcohol and tobacco use.

### Inter-rater reliability of PFI abstraction

Across data elements, between 91% and 100% of the *n* = 34 extractions were concordant (i.e., data extractions matched perfectly; Supplementary Table S1). Items with lower concordance were as follows: cause of death 55% and speech limitations 85%.

## Discussion

The LETBI PFI provides a comprehensive, feasible, and acceptable method for obtaining ante-mortem data required for clinical characterization of decedents in clinicopathologic TBI studies. Our findings suggest the PFI is a complementary source of information that can supplement the data available in an MR. Our preliminary analyses indicate a high level of concordance in PFI data extractions, supporting its utility as a reliable tool for quantitative data collection. The information provided in the PFI is crucial for characterizing the clinical signatures of PTND and understanding possible risk factors. By providing comprehensive clinical data for clinicopathological correlations, PFIs are also an important tool in ultimately identifying high-priority targets for treatment and prevention of PTND.

The PFI can be conducted by both clinicians with specialized training and non-clinical research staff. Clinical training in psychology or relevant subspecialties can facilitate rapport building and provide content expertise necessary for informed diagnostic impressions. Non-clinical research staff will likely require additional training in the sensitive management of topics such as bereavement, trauma, and addiction. It may also be beneficial to increase the number of interviews listened to or shadowed and ensure exposure to diverse examples of clinically complex cases. Non-clinical research staff may also prefer to conduct their first one to two interviews with a senior clinician present.

The type of informant can impact the information provided in a PFI, with each having relative benefits for the richness of data captured at varied life stages of the decedent. In general, we find that early life is usually best captured by parents, with later life best captured by partners or adult children for the final years prior to death. Importantly, for some decedents, friends or teammates may be more informative than family members. It is important, where possible, for the decedent to nominate the person they feel would best represent the course of their illness and their broader life experience. Multiple informants may be required if it becomes apparent when interviewing family members that there are gaps in the history they can provide.

In our experience, there are advantages and drawbacks to completing interviews both earlier and later after death, and the choice is best left to the informant. Although for many informants recall accuracy may be optimized early after injury, for some informants time permits further information gathering (e.g., from discussions with family and friends and police reports) and also affords critical time to process bereavement and reflect on clinically relevant changes that were less obvious immediately after death. The opportunity to grieve can also permit greater objectivity, facilitating descriptions of more challenging aspects of the decedent’s personality or behavior. Although the weeks to months post-death are a time of intense emotion for many informants, completing an interview can support the grieving process by inviting reminiscence and reflection. Overall, our team found that irrespective of time since death and emotionality during interviews, most informants experienced the PFI positively. Many found catharsis in discussing the life and death of their loved one and were comforted knowing that their contributions may advance research and improve outcomes for others.

Our findings suggest the PFI is a complementary source of information that can supplement the MR. The PFI was more likely to include key social determinants of health (e.g., race, ethnicity, and years of education) and family history of medical and psychiatric conditions, which are informative for building prognostication models for PTND. The PFI also provided greater detail about a decedent’s trajectories of specific clinical symptoms, including more granular descriptions of impairments in cognition, neurobehavior, and motor function. This information is critical to facilitate clinical characterization of PTND required for clinical diagnosis. Finally, the PFI was more likely to include detailed information about the cause and manner of death, including longstanding contributors to death. While this may offer unique insights into how remote TBI and PTND contribute to death, the low concordance in PFI-coded causes suggests that access to extensive contextual information about factors contributing to or preceding death may obscure the precise cause of death. On the contrary, the immediate cause of death recorded on death certificates commonly overlooks or omits important contributing factors.^61^ This underscores the value of using both the PFI and MR together.

The PFI is not without limitations. As with all retrospective data collection, the PFI can be impacted by recall bias, and it should not be assumed that all non-missing data are accurate. The PFI takes 2–3 h, which may not be feasible in all research settings. Our reflections on how informant type (e.g., parent and spouse) may have impacted data obtained were drawn from observations across decedents rather than within decedents. Finally, we did not examine the validity of PFI; however, such examinations have been discouraged in the absence of a gold standard ante-mortem data tool to define accuracy.

We have provided the measures included in our PFI to facilitate replication, expansion, and refinement of PFI in other TBI brain donor programs. We hope that this will encourage other groups working with precious brain tissue from chronic TBI survivors to implement PFI as part of a multi-modal autopsy; this holds great promise to accelerate our understanding of PTND and direct us to promising treatment targets.

## Transparency, Rigor, and Reproducibility

The analysis was conducted between October 2023 and June 2024. Data were collected using RedCap. Data were downloaded into a csv file and reviewed in the Excel program. The key inclusion criteria for entry into the parent LETBI study are established standards in the field. The outcome measures used in the PFI are established standards in the field. No statistical tests were conducted. Data are available from the primary author upon reasonable request.

## Abbreviations Used

ACT: Adult Changes in Thought
AD: Alzheimer’s disease
ADL: activities of daily living
ALS: amyotrophic lateral sclerosis
AMS: altered mental status
ASSIST: Alcohol, Smoking and Substance Involvement Screening Test
BISQ: Brain Injury Screening Questionnaire
CDE: common data elements
IADL: instrumental activities of daily living
IPV: intimate partner violence
LETBI: Late Effects of TBI study
LGBTQIA+: Lesbian, Gay, Bisexual, Transgender, Queer or Questioning, Intersex, Asexual (or Aromantic/Agender), and others.
LOC: loss of consciousness
MIDUS: Midlife in the United States
MMS: mixed martial arts
MR: medical record
NACC: National Alzheimer’s Coordinating Center
NIH: National Institutes of Health
NINDS: National Institute of Neurological Disorders and Stroke
OSA: obstructive sleep apnea
PFI: post-mortem family interview
PTD: prior to death
PTND: post-traumatic neurodegeneration
REGARDS: Reasons for Geographic and Racial Differences in Stroke
REM: rapid eye movement
RHI: repetitive head impacts
TBI: traumatic brain injury
TBIMS: Traumatic Brain Injury Model Systems
UNITE: Understanding Neurologic Injury and Traumatic Encephalopathy
WHO: World Health Organization
YPTD: years prior to death

## References

[1] Dams-O’Connor K, Juengst SB, Bogner J, et al. Traumatic brain injury as a chronic disease: Insights from the United States Traumatic Brain Injury Model Systems Research Program. Lancet Neurol 2023;22(6):517–528. 37086742 10.1016/S1474-4422(23)00065-0

[2] Brett BL, Temkin N, Barber JK, et al.; for TRACK-TBI Investigators. Long-term multidomain patterns of change after traumatic brain injury: A TRACK-TBI LONG study. Neurology 2023;101(7):e740–e753. 37344231 10.1212/WNL.0000000000207501 PMC10437015

[3] Del Pozzo J, Spielman LA, Yew B, et al. Detecting and predicting cognitive decline in individuals with traumatic brain injury: A longitudinal telephone-based study. J Neurotrauma 2024;41(15–16):1937–1947; doi: 10.1089/neu.2023.0589 38907691 PMC11564846

[4] Himanen L, Portin R, Isoniemi H, et al. Longitudinal cognitive changes in traumatic brain injury: A 30-year follow-up study. Neurology 2006;66(2):187–192. 16434651 10.1212/01.wnl.0000194264.60150.d3

[5] Baguley IJ, Cooper J, Felmingham K. Aggressive behavior following traumatic brain injury: How common is common? J Head Trauma Rehabil 2006;21(1):45–56. 16456391 10.1097/00001199-200601000-00005

[6] Crane PK, Gibbons LE, Dams-O’Connor K, et al. Association of Traumatic Brain Injury with late-life neurodegenerative conditions and neuropathologic findings. JAMA Neurol 2016;73(9):1062–1069. 27400367 10.1001/jamaneurol.2016.1948 PMC5319642

[7] Forslund MV, Perrin PB, Røe C, et al. Global outcome trajectories up to 10 years after moderate to severe traumatic brain injury. Front Neurol 2019;10:219. 30923511 10.3389/fneur.2019.00219 PMC6426767

[8] Graham N, Sharp DJ. Understanding neurodegeneration after traumatic brain injury: From mechanisms to clinical trials in dementia. J Neurol Neurosurg Psychiatry 2019;90(11):1221–1233. 31542723 10.1136/jnnp-2017-317557 PMC6860906

[9] Dams-O’Connor K. Emerging topics Focus Area 1: TBI and Alzheimer’s disease/Alzheimer’s disease related dementias (AD/ADRD) risk. Alzheimer’s Disease-Related Dementias Summit 2019: Research Challenges and Opportunities, National Institutes of Health: Bethesda, MD.; 2019.

[10] Dams-O’Connor K. Methodological challenges and opportunities in the study of TBI as a risk factor for Dementia investigation of long-term outcomes of traumatic brain injury: Statistical, psychometric and empirical methodologies. Traumatic Brain Injury as a Risk Factor for Dementia. National Institutes of Health and Veterans Administration Workshop: Bethesda, MD; 2019.

[11] Dams-O’Connor K, Awwad HO, Hoffman S, et al. Alzheimer’s disease-related dementias summit 2022: National Research priorities for the investigation of post-traumatic brain injury Alzheimer’s disease and related dementias. J Neurotrauma 2023;40(15–16):1512–1523; doi: 10.1089/neu.2022.0514 36927167 PMC10494902

[12] Dams-O’Connor K, Bellgowan PSF, Corriveau R, et al. Alzheimer’s disease-related dementias summit 2019: National Research priorities for the investigation of traumatic brain injury as a risk factor for Alzheimer’s disease and related dementias. J Neurotrauma 2021;38(23):3186–3194. 34714152 10.1089/neu.2021.0216 PMC8820282

[13] Graham NSN, Cole JH, Bourke NJ, et al. Distinct patterns of neurodegeneration after TBI and in Alzheimer’s disease. Alzheimers Dement 2023;19(7):3065–3077. 36696255 10.1002/alz.12934 PMC10955776

[14] Rost N (Scientific Chair). ADRD Summit 2022 Report. National Advisory Neurological Disorders and Stroke Council; 2022.

[15] Iacono D, Lee P, Edlow BL, et al. Early-onset dementia in war veterans: Brain polypathology and clinicopathologic complexity. J Neuropathol Exp Neurol 2020;79(2):144–162. 31851313 10.1093/jnen/nlz122 PMC6970453

[16] Agrawal S, Leurgans SE, James BD, et al. Association of traumatic brain injury with and without loss of consciousness with neuropathologic outcomes in community-dwelling older persons. JAMA Netw Open 2022;5(4):e229311. 35476062 10.1001/jamanetworkopen.2022.9311 PMC9047640

[17] Postupna N, Rose SE, Gibbons LE, et al. The delayed neuropathological consequences of traumatic brain injury in a community-based sample. Front Neurol 2021;12:624696. 33796061 10.3389/fneur.2021.624696 PMC8008107

[18] Gibbons LE, Power MC, Walker RL, et al. Association of traumatic brain injury with late life neuropathological outcomes in a community-based cohort. J Alzheimers Dis 2023;93(3):949–961. 37125552 10.3233/JAD-221224 PMC10860614

[19] Sayed N, Culver C, Dams-O’Connor K, et al. Clinical phenotype of dementia after traumatic brain injury. J Neurotrauma 2013;30(13):1117–1122. 23374007 10.1089/neu.2012.2638 PMC3705947

[20] Dams-O’Connor K, Spielman L, Hammond FM, et al. An exploration of clinical dementia phenotypes among individuals with and without traumatic brain injury. NeuroRehabilitation 2013;32(2):199–209. 23535782 10.3233/NRE-130838 PMC3767178

[21] Adams JH, Graham DI, Murray LS, et al. Diffuse axonal injury due to nonmissile head injury in humans: An analysis of 45 cases. Ann Neurol 1982;12(6):557–563. 7159059 10.1002/ana.410120610

[22] Adams JH, Doyle D, Graham DI, et al. Diffuse axonal injury in head injuries caused by a fall. Lancet 1984;2(8417–8418):1420–1422. 6151042 10.1016/s0140-6736(84)91620-9

[23] Adams JH, Doyle D, Graham DI, et al. Microscopic diffuse axonal injury in cases of head injury. Med Sci Law 1985;25(4):265–269; doi: 10.1177/002580248502500407 4068956

[24] Roberts GW, Gentleman SM, Lynch A, et al. βA4 amyloid protein deposition in brain after head trauma. Lancet 1991;338(8780):1422–1423. 1683421 10.1016/0140-6736(91)92724-g

[25] Gentleman SM, Nash MJ, Sweeting CJ, et al. β-Amyloid precursor protein (βAPP) as a marker for axonal injury after head injury. Neurosci Lett 1993;160(2):139–144. 8247344 10.1016/0304-3940(93)90398-5

[26] Sherriff FE, Bridges LR, Gentleman SM, et al. Markers of axonal injury in post mortem human brain. Acta Neuropathol 1994;88(5):433–439. 7847072 10.1007/BF00389495

[27] Sherriff FE, Bridges LR, Sivaloganathan S. Early detection of axonal injury after human head trauma using immunocytochemistry for beta-amyloid precursor protein. Acta Neuropathol 1994;87(1):55–62. 8140894 10.1007/BF00386254

[28] McKenzie JE, Gentleman SM, Roberts GW, et al. Increased numbers of beta APP-immunoreactive neurones in the entorhinal cortex after head injury. Neuroreport 1994;6(1):161–164. 7703405 10.1097/00001756-199412300-00041

[29] Graham DI, Gentleman SM, Lynch A, et al. Distribution of beta-amyloid protein in the brain following severe head injury. Neuropathol Appl Neurobiol 1995;21(1):27–34. 7770117 10.1111/j.1365-2990.1995.tb01025.x

[30] Nicoll JA, Roberts GW, Graham DI. Apolipoprotein E epsilon 4 allele is associated with deposition of amyloid beta-protein following head injury. Nat Med 1995;1(2):135–137. 7585009 10.1038/nm0295-135

[31] McKenzie KJ, McLellan DR, Gentleman SM, et al. Is beta-APP a marker of axonal damage in short-surviving head injury? Acta Neuropathol 1996;92(6):608–613. 8960319 10.1007/s004010050568

[32] Smith DH, Chen X-H, Iwata A, et al. Amyloid beta accumulation in axons after traumatic brain injury in humans. J Neurosurg 2003;98(5):1072–1077. 12744368 10.3171/jns.2003.98.5.1072

[33] Smith C, Graham DI, Murray LS, et al. Tau immunohistochemistry in acute brain injury. Neuropathol Appl Neurobiol 2003;29(5):496–502. 14507341 10.1046/j.1365-2990.2003.00488.x

[34] Edlow BL, Keene CD, Perl DP, et al. Multimodal characterization of the late effects of traumatic brain injury: A methodological overview of the late effects of traumatic brain injury project. J Neurotrauma 2018;35(14):1604–1619. 29421973 10.1089/neu.2017.5457 PMC6016096

[35] Mez J, Solomon TM, Daneshvar DH, et al. Assessing clinicopathological correlation in chronic traumatic encephalopathy: Rationale and methods for the UNITE study. Alzheimers Res Ther 2015;7(1):62. 26455775 10.1186/s13195-015-0148-8 PMC4601147

[36] Anonymous. Chronic TBI-Related Neurodegeneration CDEs. n.d. Available from: https://fitbir.nih.gov/chronic-tbi-related-neurodegeneration-cdes [Last accessed: June 20, 2024].

[37] Au R, Seshadri S, Knox K, et al. The Framingham brain donation program: Neuropathology along the cognitive continuum. Curr Alzheimer Res 2012;9(6):673–686. 22471865 10.2174/156720512801322609 PMC3622706

[38] Davis PB, White H, Price JL, et al. Retrospective postmortem dementia assessment. Validation of a new clinical interview to assist neuropathologic study. Arch Neurol 1991;48(6):613–617. 2039384 10.1001/archneur.1991.00530180069019

[39] Rockwood K, Howard K, Thomas VS, et al. Retrospective diagnosis of dementia using an informant interview based on the Brief Cognitive Rating Scale. Int Psychogeriatr 1998;10(1):53–60. 9629524 10.1017/s1041610298005146

[40] Ellis RJ, Jan K, Kawas C, et al. Diagnostic validity of the dementia questionnaire for Alzheimer disease. Arch Neurol 1998;55(3):360–365. 9520010 10.1001/archneur.55.3.360

[41] Werlang BG, Botega NJ. A semi-structured interview for psychological autopsy in suicide cases. Braz J Psychiatry 2003;25(4):212–219. 15328546 10.1590/s1516-44462003000400006

[42] Henry M, Greenfield BJ. Therapeutic effects of psychological autopsies. Crisis 2009;30(1):20–24. 19261564 10.1027/0227-5910.30.1.20

[43] Cavanagh JTO, Carson AJ, Sharpe M, et al. Psychological autopsy studies of suicide: A systematic review. Psychol Med 2003;33(3):395–405. 12701661 10.1017/s0033291702006943

[44] Bolger JP, Strauss ME, Kennedy JS. Feasibility of retrospective assessments of behavioral symptoms in Alzheimer’s disease: A preliminary study of postmortem caregiver reports. Int Psychogeriatr 1998;10(1):61–69. 9629525 10.1017/s1041610298005158

[45] Kelly TM, Mann JJ. Validity of DSM-III-R diagnosis by psychological autopsy: A comparison with clinician ante-mortem diagnosis. Acta Psychiatr Scand 1996;94(5):337–343. 9124080 10.1111/j.1600-0447.1996.tb09869.x

[46] Chandramohan D, Fottrell E, Leitao J, et al. Estimating causes of death where there is no medical certification: Evolution and state of the art of verbal autopsy. Glob Health Action 2021;14(Suppl 1):1982486. 35377290 10.1080/16549716.2021.1982486 PMC8986278

[47] Soleman N, Chandramohan D, Shibuya K. Verbal autopsy: Current practices and challenges. Bull World Health Organ 2006;84(3):239–245. 16583084 10.2471/blt.05.027003 PMC2627297

[48] Gouda HN, Kelly-Hanku A, Wilson L, et al. “Whenever they cry, I cry with them”: Reciprocal relationships and the role of ethics in a verbal autopsy study in Papua New Guinea. Soc Sci Med 2016;163:1–9. 27376593 10.1016/j.socscimed.2016.06.041

[49] Fottrell E, Byass P. Verbal autopsy: Methods in transition. Epidemiol Rev 2010;32:38–55. 20203105 10.1093/epirev/mxq003

[50] Mikkelsen L, Phillips DE, AbouZahr C, et al. A global assessment of civil registration and vital statistics systems: Monitoring data quality and progress. Lancet 2015;386(10001):1395–1406. 25971218 10.1016/S0140-6736(15)60171-4

[51] Yang G, Rao C, Ma J, et al. Validation of verbal autopsy procedures for adult deaths in China. Int J Epidemiol 2006;35(3):741–748. 16144861 10.1093/ije/dyi181

[52] Katz DI, Bernick C, Dodick DW, et al. National Institute of Neurological disorders and Stroke consensus diagnostic criteria for traumatic encephalopathy syndrome. Neurology 2021;96(18):848–863. 33722990 10.1212/WNL.0000000000011850 PMC8166432

[53] Howard VJ, Cushman M, Pulley L, et al. The reasons for geographic and racial differences in stroke study: Objectives and design. Neuroepidemiology 2005;25(3):135–143. 15990444 10.1159/000086678

[54] Brim OG, Ryff CD, Kessler RC. How Healthy Are We?: A National Study of Well-Being at Midlife. University of Chicago Press; 2004.

[55] Beekly DL, Ramos EM, van Belle G, et al. The National Alzheimer’s Coordinating Center (NACC) database: An Alzheimer disease database. Alzheimer Dis Assoc Disord 2004;18(4):270–277. 15592144

[56] D’Ambruoso L, Byass P, Qomariyah SN, et al. A lost cause? Extending verbal autopsy to investigate biomedical and socio-cultural causes of maternal death in Burkina Faso and Indonesia. Soc Sci Med 2010;71(10):1728–1738. 20646807 10.1016/j.socscimed.2010.05.023

[57] Alencar Albuquerque G, de Lima Garcia C, da Silva Quirino G, et al. Access to health services by lesbian, gay, bisexual, and transgender persons: Systematic literature review. BMC Int Health Hum Rights 2016;16:2. 26769484 10.1186/s12914-015-0072-9 PMC4714514

[58] Hashemi G, Wickenden M, Bright T, et al. Barriers to accessing primary healthcare services for people with disabilities in low and middle-income countries, a meta-synthesis of qualitative studies. Disabil Rehabil 2022;44(8):1207–1220. 32956610 10.1080/09638288.2020.1817984

[59] Clemente KAP, da Silva SV, Vieira GI, et al. Barriers to the access of people with disabilities to health services: A scoping review. Rev Saude Publica 2022;56:64. 35792776 10.11606/s1518-8787.2022056003893 PMC9239543

[60] Fottrell E. Dying to count: Mortality surveillance in resource-poor settings. Glob Health Action 2009;2:10.3402/gha.v2i0.1926; doi: 10.3402/gha.v2i0.1926 PMC2779939 20027269

[61] Betz ME, Kelly SP, Fisher J. Death certificate inaccuracy and underreporting of injury in elderly people. J Am Geriatr Soc 2008;56(12):2267–2272. 19093926 10.1111/j.1532-5415.2008.02001.x

[62] Hoff CJB, Ratard R. Louisiana death certificate accuracy: A concern for the public’s health. J La State Med Soc 2010;162(6):350–353. 21294493

[63] Halanych JH, Shuaib F, Parmar G, et al. Agreement on cause of death between proxies, death certificates, and clinician adjudicators in the Reasons for Geographic and Racial Differences in Stroke (REGARDS) study. Am J Epidemiol 2011;173(11):1319–1326. 21540327 10.1093/aje/kwr033 PMC3101067

[64] Dewan MC, Rattani A, Gupta S, et al. Estimating the global incidence of traumatic brain injury. J Neurosurg 2019;130(4):1080–1097. 29701556 10.3171/2017.10.JNS17352

[65] Monahan K. Intimate partner violence and traumatic brain injury: A public health issue. J Neurol Neuromed 2018;3(3):3–6.

[66] Whiteneck GG, Cuthbert JP, Corrigan JD, et al. Prevalence of self-reported lifetime history of traumatic brain injury and associated disability: A statewide population-based survey. J Head Trauma Rehabil 2016;31(1):E55–E62. 25931187 10.1097/HTR.0000000000000140

[67] Dams-OConnor K, Cantor JB, Brown M, et al. Screening for traumatic brain injury: Findings and public health implications. J Head Trauma Rehabil 2014;29(6):479–489. 25370440 10.1097/HTR.0000000000000099 PMC4985006

[68] Seichepine DR, Stamm JM, Daneshvar DH, et al. Profile of self-reported problems with executive functioning in college and professional football players. J Neurotrauma 2013;30(14):1299–1304. 23421745 10.1089/neu.2012.2690 PMC3713446

[69] Anonymous. Home - REGARDS Study. n.d. Available from: https://www.uab.edu/soph/regardsstudy/ [Last accessed: November 19, 2024].

[70] Anonymous. MIDUS - Midlife in the United States, A National Longitudinal Study of Health and Well-Being. n.d. Available from: https://midus.wisc.edu/ [Last accessed: November 19, 2024].

[71] Anonymous. NACC Home. n.d. Available from: https://naccdata.org/ [Last accessed: November 19, 2024].

[72] Anonymous. UNITE Study. n.d. Available from: https://www.bu.edu/alzresearch/ctecenter/cte-center-research/unite-study/ [Last accessed: November 19, 2024].

[73] McNair DM, Kahn RJ. Self-assessment of cognitive deficits. In: Assessment in geriatric psychopharmacology, vol. 137. (Crook T, Ferris S, Bartus R., Eds.) Mark Powley: New Canaan, CT; 1983, p. 143.

[74] Jorm AF, Korten AE. Assessment of cognitive decline in the elderly by informant interview. Br J Psychiatry 1988;152:209–213. 3167337 10.1192/bjp.152.2.209

[75] Cummings JL, Mega M, Gray K, Rosenberg-Thompson S, Carusi DA, Gornbein J. The Neuropsychiatric Inventory: comprehensive assessment of psychopathology in dementia. Neurology 1994;44(12):2308–2314. 7991117 10.1212/wnl.44.12.2308

[76] Cummings J. The Neuropsychiatric Inventory: Development and Applications. J Geriatr Psychiatry Neurol 2020;33(2):73–84. 32013737 10.1177/0891988719882102 PMC8505128

[77] Anonymous. TBINDSC. n.d. Available from: https://www.tbindsc.org/ [Last accessed: November 19, 2024].

[78] Boeve BF, Molano JR, Ferman TJ, et al. Validation of the Mayo Sleep Questionnaire to screen for REM sleep behavior disorder in an aging and dementia cohort. Sleep Med 2011;12(5):445–453. 21349763 10.1016/j.sleep.2010.12.009 PMC3083495

[79] Anonymous. ACT Study Home. n.d. Available from: https://actagingresearch.org/ [Last accessed: November 19, 2024].

[80] Cedarbaum JM, Stambler N, Malta E, et al. The ALSFRS-R: a revised ALS functional rating scale that incorporates assessments of respiratory function. BDNF ALS Study Group (Phase III). J Neurol Sci 1999;169(1–2):13–21. 10540002 10.1016/s0022-510x(99)00210-5

[81] Bayliss EA, Ellis JL, Steiner JF. Seniors’ self-reported multi-morbidity captured biopsychosocial factors not incorporated into two other data-based morbidity measures. Journal of Clinical Epidemiology 2009;62(5):550–557.e1. 18757178 10.1016/j.jclinepi.2008.05.002 PMC2743235

[82] Poitras ME, Fortin M, Hudon C, Haggerty J, Almirall J. Validation of the disease burden morbidity assessment by self-report in a French-speaking population. BMC Health Serv Res 2012;12(1):35. 22333434 10.1186/1472-6963-12-35 PMC3305524

[83] Ware JE, Jr, Sherbourne CD. The MOS 36-ltem Short-Form Health Survey (SF-36): I. Conceptual Framework and Item Selection. Med Care 1992;30(6):473. 1593914

[84] Reisberg B. Functional assessment staging (FAST). Psychopharmacol Bull 1988;24(4):653–659. 3249767

[85] WHO ASSIST Working Group. The Alcohol, Smoking and Substance Involvement Screening Test (ASSIST): development, reliability and feasibility. Addiction 2002;97(9):1183–1194. 12199834 10.1046/j.1360-0443.2002.00185.x

[86] Brown GL, Goodwin FK, Ballenger JC, Goyer PF, Major LF. Aggression in humans correlates with cerebrospinal fluid amine metabolites. Psychiatry Res 1979;1(2):131–139. 95232 10.1016/0165-1781(79)90053-2

[87] Beekly DL, Ramos EM, Lee WW, et al. The National Alzheimer’s Coordinating Center (NACC) database: The uniform data set. Alzheimer Dis Assoc Disord 2007;21(3):249–258. 17804958 10.1097/WAD.0b013e318142774e

[88] Dijkers MP, Harrison-Felix C, Marwitz JH. The traumatic brain injury model systems: History and contributions to clinical service and research. J Head Trauma Rehabil 2010;25(2):81–91. 20134334 10.1097/HTR.0b013e3181cd3528

[89] Maas AI, Harrison-Felix CL, Menon D, et al. Common data elements for traumatic brain injury: Recommendations from the interagency working group on demographics and clinical assessment. Arch Phys Med Rehabil 2010;91(11):1641–1649. 21044707 10.1016/j.apmr.2010.07.232

