# Supplementary material for: Ante-Mortem Clinical Characterization with Post-Mortem Family Interview and Medical Record Abstraction in a Traumatic Brain Injury Brain Donor Program
Source: Neurotrauma Rep. 2025 Aug 5;6(1):624–37. doi: 10.1177/08977151251362180 (PMC12413259; doi:10.1177/08977151251362180)
Supplement: Supplementary Table S1 [file 08977151251362180_supplementary_table_s1.docx]

**Supplementary Table 1**

*Postmortem Family Interview: Concordance Between Two Data Extractors for n=34 decedents*

|  | Concordance n (%) |
| --- | --- |
| DEMOGRAPHICS | |
| Date of Birth | 34 (100%) |
| Multiple Birth | 33 (97%) |
| Date of Death | 34 (100%) |
| Gender | 34 (100%) |
| Race | 33 (97%) |
| Ethnicity | 32 (94%) |
| Marital Status | 32 (94%) |
| Residence | 33 (97%) |
| Type of Residence | 33 (97%) |
| Primary Caregiver | 34 (100%) |
| Subject Lived With | 33 (97%) |
| English as Primary Language | 34 (100%) |
| Country of Birth | 32 (94%) |
| Years of Education | 34 (100%) |
| Primary Occupation | 33 (97%) |
| DETAILS OF DEATH | |
| Cause of Death | 18 (55%) |
| Place of Death | 32 (94%) |
| MEDICAL HEALTH & MEDICATIONS | |
| Surgeries YPTD | 34 (100%) |
| Hospitalizations YPTD | 34 (100%) |
| Medications YPTD | 34 (100%) |
| FAMILY MEDICAL & PSYCHIATRIC HISTORY | |
| Memory problems | 33 (97%) |
| Developmental Disability | 33 (97%) |
| Neurological Disorders | 33 (97%) |
| Psychiatric Problems | 33 (97%) |
| CLINICAL FUNCTION | |
| Decline in clinical function noted by a clinician | 34 (100%) |
| Overall course of clinical decline | 33 (97%) |
| Predominant domain of impairment/ decline | 32 (94%) |
| CLINICAL FUNCTION – COGNITIVE DYSFUNCTION | |
| Memory impairment present | 34 (100%) |
| Remembered things about family and friends (e.g. occupations, birthdays) | 33 (97%) |
| Remembered things that happened recently (e.g. friends visiting) | 33 (97%) |
| Recalled conversations from a few days earlier | 33 (97%) |
| Remembered their address/ phone number | 33 (97%) |
| Remembered the day and month | 32 (94%) |
| Remembered where things were usually kept (e.g. milk is in the refrigerator) | 33 (97%) |
| Remembered where to find things which had been put in a different place than usual | 33 (97%) |
| Knew how to work familiar machines around the house (e.g., the toaster) | 32 (94%) |
| Learned how to use a new gadget or machine around the house | 31 (91%) |
| Learned new things in general | 32 (94%) |
| Executive function impairment present | 34 (100%) |
| Made decisions on everyday matters | 33 (97%) |
| Handled other everyday arithmetic problems (e.g. knew how much food to buy) | 32 (94%) |
| Used his/her intelligence to understand what was going on and to reason things out | 31 (91%) |
| Language impairment present | 32 (94%) |
| Ability to speak limited (1 to 5 words a day) | 29 (85%) |
| All intelligible vocabulary lost | 32 (94%) |
| Visuospatial function impairment present | 34 (100%) |
| Attention impairment present | 34 (100%) |
| Followed a story in a book or on TV | 33 (97%) |
| Fluctuating cognition present | 32 (94%) |
| Predominant cognitive symptom | 33 (97%) |
| Mode of onset of cognitive symptoms | 33 (97%) |
| CLINICAL FUNCTION – NEUROBEHAVIORUAL & EMOTIONAL DYSFUNCTION | |
| Apathy | 33 (97%) |
| Depression | 33 (97%) |
| Visual hallucinations | 34 (100%) |
| Auditory hallucinations | 33 (97%) |
| Disinhibition | 33 (97%) |
| Irritability | 33 (97%) |
| Agitation | 34 (100%) |
| Personality change | 33 (97%) |
| REM sleep behaviour disorder | 33 (97%) |
| Anxiety | 29 (85%) |
| Predominant neurobehavioral/emotional symptom | 34 (100%) |
| Mode of onset of neurobehavioral/emotional symptom | 32 (94%) |
| CLINICAL FUNCTION – MOTOR IMPAIRMENT | |
| Gait disorder | 33 (97%) |
| Falls | 33 (97%) |
| Tremor | 33 (97%) |
| Slowness | 34 (100%) |
| Predominant motor symptom | 33 (97%) |
| Mode of onset of motor symptom | 33 (97%) |
| Lifting or carrying groceries | 33 (97%) |
| Bathing or dressing themselves | 33 (97%) |
| Climbing several flights of stairs | 32 (94%) |
| Climbing one flight of stairs | 32 (94%) |
| Bending, kneeling or stooping | 32 (94%) |
| Walking more than 1 mile | 32 (94%) |
| Walking several blocks | 32 (94%) |
| Walking 1 block | 32 (94%) |
| Vigorous activity (e.g. running) | 32 (94%) |
| Moderate activity (e.g. bowling) | 32 (94%) |
| Nonambulatory | 31 (91%) |
| Unable to sit up independently | 31 (91%) |
| Unable to smile | 33 (97%) |
| Unable to hold head up | 33 (97%) |
| CLINICAL FUNCTION – FUNCTIONAL IMPAIRMENT | |
| Urinary Incontinence | 32 (94%) |
| Fecal Incontinence | 32 (94%) |
| Simple financial management (e.g. paying bills) | 33 (97%) |
| Handled money for shopping | 33 (97%) |
| Complex financial management (e.g. tax filing) | 33 (97%) |
| Handled financial matters (e.g. their pension, dealings with the bank) | 32 (94%) |
| Shopping | 33 (97%) |
| Hobbies and skilled games (e.g. chess) | 32 (94%) |
| Basic household tasks (e.g. make a cup of coffee) | 34 (100%) |
| More complex household tasks (e.g. cooking) | 33 (97%) |
| Tracking current events | 33 (97%) |
| Understanding TV/book | 33 (97%) |
| Remembering appointments/ holidays/ medications | 34 (100%) |
| Travelling outside neighbourhood – driving/public transport | 34 (100%) |
| Decreased ability to perform complex tasks (e.g., planning dinner for guests) | 34 (100%) |
| Requires assistance in choosing proper clothing | 33 (97%) |
| Difficulty putting clothing on properly | 33 (97%) |
| SUBSTANCE USE | |
| Tobacco products | 34 (100%) |
| Alcoholic beverages | 33 (97%) |
| Cannabis | 32 (94%) |
| Cocaine | 33 (97%) |
| Amphetamine type stimulants | 33 (97%) |
| Inhalants | 33 (97%) |
| Sedatives or Sleeping Pills | 34 (100%) |
| Hallucinogens | 33 (97%) |
| Opioids | 33 (97%) |

Note. An item was considered concordant when, based on open text fields and structured questionnaire responses, both data abstractors extracted the exact same data for a given data element or where both team members agreed the data point was missing. For Likert scale responses, such as those grading the severity of clinical dysfunction, an exact match for each data element was required (e.g. both abstractors agreed there had been a ‘minor decrease’ in function). Open text fields also required an exact match (e.g. date of birth and date of death had to match day, month, and year). This concordance analysis includes missing data. The denominator for every cell is n=34. PFI – postmortem family interview, YPTD – year prior to death.
